# Rifampicin in Nontuberculous Mycobacterial Infections: Acute Kidney Injury with Hemoglobin Casts

**DOI:** 10.1155/2018/9321621

**Published:** 2018-04-05

**Authors:** Rishi Kora, Sergey V. Brodsky, Tibor Nadasdy, Dean Agra, Anjali A. Satoskar

**Affiliations:** ^1^Mount Carmel West Hospital, Columbus, OH, USA; ^2^Ohio State University Wexner Medical Center, Columbus, OH, USA; ^3^Columbus Nephrology, Columbus, OH, USA

## Abstract

Rifampicin is a key component of multidrug regimens not only for tuberculosis, but also nontuberculous mycobacterial infections (NTM) which are on the rise worldwide. Knowledge of the toxicity profile is important. Hepatotoxicity is a well-known side effect of Rifampicin necessitating regular liver function monitoring during therapy. Acute kidney injury (AKI) is a relatively rare complication, usually resulting from allergic interstitial nephritis (AIN). Rifampicin-induced intravascular hemolysis resulting in hemoglobinuria and AKI is even more uncommon, especially in Western countries with low prevalence of mycobacterial infections. Rifampicin-induced antibodies are implicated and this complication preferentially occurs during intermittent drug treatment protocols or when Rifampicin is restarted after a long drug-free interval. Awareness of this drug complication and its unique timing is important especially among emergency room physicians where patients with AKI may first present. It is equally important for nephrologists and pathologists. We describe one such case with detailed clinical course of the patient and interesting biopsy findings of ATN with intratubular hemoglobin casts.

## 1. Introduction

This is a case of an elderly female with recurrent Mycobacterium avium-intracellulare (MAI) pneumonia, who was restarted on Rifampicin-containing triple drug regimen (after a 16-month drug-free interval) and presented with acute kidney injury (AKI) and blood in the urine ten days after starting treatment with Rifampicin. Serum creatinine was normal before presentation and there was no history of other nephrotoxic insults. A kidney biopsy was performed to identify the etiology of AKI and to look for potentially treatable causes.

## 2. Case

### 2.1. Clinical History

Patient is a 65-year-old Caucasian female with past medical history of hypothyroidism and gastroesophageal reflux disease. MAI pneumonia was diagnosed two years ago based on symptoms of shortness of breath, wheezing, and mild hemoptysis; chest CT findings of diffuse bilateral centrilobular nodular ground glass opacities; and bronchoalveolar lavage (BAL) fluid cultures revealing MAI complex. Lung biopsy showed chronic bronchiolitis and rare nonnecrotizing granulomas. She was started on a three-drug regimen with Azithromycin (600 mg), Ethambutol (1800 mg), and Rifampicin (600 mg) three times per week [1]. Patient reported adherence and tolerance to her therapeutic regimen for 8 months. After that, she stopped treatment because of insurance issues.

Sixteen months later, she was found to have recurrent MAI pneumonia, confirmed on bronchoscopy and BAL fluid culture. She was resumed on the same regimen of Azithromycin, Ethambutol, and Rifampicin. Ten days later, she developed nausea, vomiting, weakness, fever, diarrhea, and decreasing urine output.

**Figure 1:**
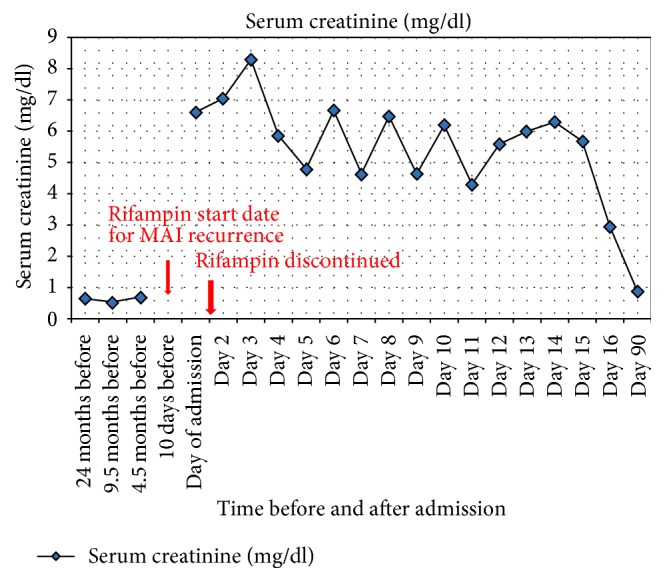
Graph with patient's serum creatinine over time, before and after admission for AKI.

On admission, she had AKI with serum creatinine of 6.6 mg/dl and blood urea nitrogen of 68 mg/dl. Baseline serum creatinine was 0.7 mg/dl three months prior to this presentation (Figure 1) and eGFR was 91 ml/min by CKD-EPI creatinine equation [2]. Laboratory results are shown in Table 1. Patient was anemic with anion gap metabolic acidosis. Urinalysis showed blood, subnephrotic proteinuria (Table 2). Renal ultrasound displayed normal size kidneys measuring 12.9 cm and 12.1 cm, without increased echogenicity, hydronephrosis, masses, or calculus. Patient denied recent use of nonsteroidal anti-inflammatory medications or recent exposure to radiographic contrast.

### 2.2. Kidney Biopsy

**Figure 2:**
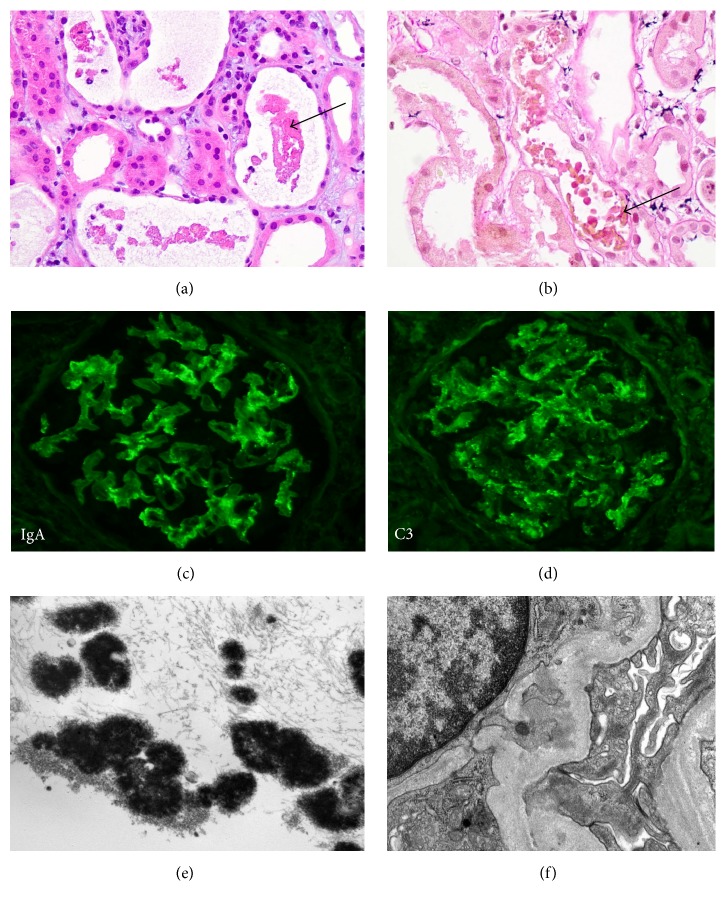
(a) Kidney biopsy showing acute tubular necrosis (ATN) with globular cast material in the tubules and interstitial edema (hematoxylin and eosin stained 200x). (b) Special stain for hemoglobin highlights yellow staining in the globular cast material (200x). ((c) and (d)) Direct immunofluorescence staining for IgA and C3 shows granular mesangial staining, respectively (400x). (e) Ultrastructural examination shows osmophilic globular hemoglobin casts in tubular lumen (uranyl acetate and lead citrate fixation, 10,000x). (f) Ultrastructural examination shows small scattered paramesangial electron dense immune-type deposits (uranyl acetate and lead citrate fixation, 12,000x).

The biopsy processed per routine protocol (light microscopy, direct immunofluorescence, and electron microscopy) had 23 glomeruli with mild focal mesangial hypercellularity, diffuse acute tubular necrosis (ATN), interstitial edema, and granular/globular casts in scattered tubules (Figure 2(a)). Immunoperoxidase stain for myoglobin was negative, but hemoglobin stain showed yellow-green staining in the globular tubular casts (Figure 2(b)). Interstitial inflammation was mild and patchy. Interstitial fibrosis and tubular atrophy were mild, involving less than 20% of the renal cortex. Direct immunofluorescence study showed mild (1+) mesangial IgA and complement C3 (Figures 2(c) and 2(d)). Electron microscopic examination showed tubules with globular casts and few scattered mesangial electron dense immune-type deposits (Figures 2(e) and 2(f)). In the absence of recent/resolving bacterial infection, the mesangial IgA immune complex deposits suggested mild underlying chronic IgA nephropathy. But there were no active proliferative glomerular lesions and the patient had normal renal function prior to this episode of AKI. Also, this may represent coincidental IgA deposits which can be seen in 3% of the population in the Western countries [3]. IgA nephropathy causes hematuria, but usually not hemoglobinuria (which this patient had). The AKI was attributed primarily to the ATN, which can be multifactorial. The presence of hemoglobin-containing tubular casts however suggested the possibility of ongoing intravascular hemolysis and hemoglobinuria as the plausible cause of the ATN. Since the patient was not receiving any other medications implicated in hemolysis, Rifampicin was considered the likely culprit [4].

### 2.3. Clinical Management and Followup

Rifampicin and the other antimycobacterial drugs were discontinued. Patient remained anuric for 3 days despite intravenous hydration followed by Lasix challenge. She was initiated on hemodialysis and underwent seven sessions during her 14-day hospital stay and five subsequent sessions as an outpatient. Antimycobacterial treatment was held for 6 weeks after discharge and subsequently resumed on Ethambutol and Azithromycin. Renal function slowly recovered to 1.94 mg/dl at one month and 0.7 mg/dl at three months (eGFR > 60 ml/minute).

## 3. Discussion

Rifampicin is used in multidrug treatment regimens not only for tuberculosis, but also for NTM infections, leprosy, and Staphylococcal infections [1, 5, 6]. Rifampicin is designated as an essential drug by the WHO [7]. Hepatotoxicity is well-known side effect and liver function monitoring during treatment is routinely performed with the use of this drug [8]. However, AKI is a less common complication of Rifampicin. Despite published reports [9–13], regular renal function monitoring is not routine practice. So patients may present unexpectedly to urgent care facilities with AKI. In this setting, it may not be so intuitive to identify Rifampicin as the trigger for the AKI. Additionally, the patient may be on several other commonly prescribed medications such as antibiotics, proton-pump inhibitors and herbal supplements that are also implicated in the causation of ATN and interstitial nephritis. All these confounding factors may preclude the correct diagnosis. Another important confounding factor is the unique temporal relation between start of Rifampicin-based therapy and the development of AKI. It usually occurs in the setting of reintroduction of therapy after a long drug-free interval and typically few weeks after the start of the day (in contrast to 24 to 48 hours in a typical allergic reaction), [5, 9–13]. Clinical symptoms frequently reported include nausea and vomiting (72%), fever (45%), chills (43%), abdominal pain (40%), diarrhea (26%), jaundice (19%), lumbar pain (17%), and anemia (96%) [12]. Duration of anuric phase is on average 11.4 days +/− 7 days. An average of 4.8 +/− 4.6 hemodialysis sessions is required [12]. Our patient displayed nearly all of the aforementioned features except jaundice.

The mechanisms of rifampicin-induced AKI may be multiple and attributed to type II or III hypersensitivity reactions. Development of anti-Rifampicin antibodies has been described [5, 12]. These antibodies however bind to Rifampicin and these immune complexes are cleared from the circulation. On daily regimens, continuous formation of antigen-antibody complexes prevents rise in free antibody titer to dangerous levels. Rise in titers however does occur with intermittent drug regimen or after prolonged discontinuation and reinstitution of treatment. Unfortunately however, testing for Rifampicin antibodies was not performed in our patient since this is not a routine laboratory test and was unlikely to alter management significantly.

Rifampicin is one of the few drugs known to cause hemolysis [4]. One way is through cross-reaction of Rifampicin antibodies with the blood group I antigen on red blood cells leading to complement-mediated hemolysis. Hemolytic anemia and positive antiglobulin test has been described during Rifampicin treatment [5]. Although antiglobin testing was not performed in our patient, the kidney biopsy did demonstrate hemoglobin-containing tubular casts. Hemoglobin casts are usually small in size, containing globular fragments, more coarse than the usual granular ATN casts, and with targetoid morphology on electron microscopy (Figure 2). They may sometimes be confused with red blood cells (RBCs); in contrast, the hemoglobin globules are uneven in size unlike RBCs. Urinalysis revealed blood on dipstick examination but not RBCs, supportive of hemoglobinuria. Additionally, the patient had low serum hemoglobin level with mildly elevated serum LDH (lactate dehydrogenase) (Table 1), supportive of intravascular hemolysis. But it was not massive hemolysis, since haptoglobin was not low. The most common histologic findings in Rifampicin-associated AKI are interstitial nephritis and associated ATN [13]. Minimal change disease has also been reported [14]. However, our case is unique in that it showed predominantly ATN with hemoglobin-containing tubular casts due to Rifampicin-induced hemolysis (not interstitial nephritis). Although hemoglobin is a normal body pigment, its presence in the tubules is toxic to the tubular epithelial cells [15].

Although Ethambutol also has been reported to cause AKI [16], it is more commonly known to cause ocular optic neuropathy [17]. Most of the published reports suggest Rifampicin as the major culprit for AKI [9–13]. Covic et al. [10] reported AKI in 60 out of 120,132 patients (0.05%) receiving Rifampicin. A much higher prevalence has been reported by Chang et al. [9] in Taiwan (7.1%) probably because of a large population of elderly patients afflicted by tuberculosis in that region. In this large case series by Chang et al., out of 1,394 patients treated with Rifampicin, 99 patients developed AKI. Rifampicin was discontinued in 34 patients. Among them, a rechallenge was performed in 21 patients, with six developing a second episode of AKI. Recovery rate following AKI was reported to be 73%.

In conclusion, our patient developed AKI following reintroduction of Rifampicin-based therapy for recurrent MAI after a prolonged drug-free period. It was followed by slow but complete renal recovery after dialysis and cessation of the drug. Rifampicin is used not only for treatment of mycobacterium tuberculosis infection, but also in nontuberculous mycobacterial infections (NTM) which are prevalent worldwide. Since there is no official recommendation to regularly monitor patient's renal function during Rifampicin therapy, patients may present unexpectedly with AKI. So awareness of renal toxicity of Rifampicin and the unique timing of occurrence is important not only among infectious disease physicians but also among emergency room physicians and nephrologists who are often consulted. It is also important to understand the different mechanisms of rifampicin-induced AKI. In AKI due to ATN alone (without interstitial nephritis), steroid therapy is not warranted in this patient. For the pathologists who evaluate the kidney biopsy, procuring detailed clinical history from the physician and attention to morphologic details in the biopsy are crucial. One should be particularly aware of this subtle histologic finding of small intratubular hemoglobin casts and understand the utility of the hemoglobin stain. This provides an important clue to identify the cause of ATN, which otherwise can be difficult, especially in the presence of several other confounding factors such as recent bacterial infections and concomitant use of other potentially nephrotoxic drugs.

## Figures and Tables

**Table 1:** Laboratory testing for serum metabolites at admission.

Comprehensive metabolic profile	Measured value	Reference value
Sodium	131 mMol/L	136–145 mMol/L
Potassium	4.4 mMol/L	3.6–5.1 mMol/L
Chloride	98 mMol/L	98–107 mMol/L
Bicarbonate	17 mMol/L	22–32 mMol/L
Anion gap	16 mMol/L	6–18 mMol/L
Glucose	84 mg/dl	70–110 mg/dl
BUN	68 mg/dl	8–20 mg/dl
Creatinine	6.61 mg/dl	0.6–1.30 mg/dl
Alkaline phosphatase	160 units/L	32–91 units/L
ALT/SGPT	19 units/L	14–63 units/L
AST/SGOT	80 units/L	15–41 units/L
Bilirubin total	2.1 mg/dl	0.3–1.2 mg/dl
Bilirubin direct	1.0 mg/dl	0.1–0.5 mg/dl
Bilirubin indirect	1.1 mg/dl	0.0–1.0 mg/dl
C3	122 mg/dl	79–152 mg/dl
C4	25 mg/dl	16–38 mg/dl
Haptoglobin	225 mg/dl	36–195 mg/dl
Lactate dehydrogenase	232 units/L	98–192 units/L
Hemoglobin	11.4 gm/dl	12–16 gm/dl

**Table 2:** Urinalysis results at admission.

Urinalysis	Measured value	Reference value
Urine color	Yellow	Yellow
Urine appearance	Turbid	Clear
Urine specific gravity	1.012	1.002–1.030
Urine pH	6	4.5–8.0
Urine glucose	Normal	Normal
Urine ketones	Negative	Negative
Urine bilirubin	Negative	Negative
Urine blood	300/UL	Negative
Urine urobilinogen	Normal	Normal
Urine leukocyte esterase	500/UL	Negative
Urine nitrite	Negative	Negative
Urine protein	200 mg/dl	Negative
WBC urine	183/hpf	0–5/hpf
RBC urine	10/hpf	0–5/hpf
Squamous epithelial cells urine	Moderate	Few/hpf
Transitional epithelial cells urine	Few	None/hpf
Urine bacteria	Moderate	None/hpf
Urine mucous	Rare	None/lpf
Microalbumin creatinine ratio	1300 mg/gram	<30 mg/gram
Protein/creatinine ratio	2400 mg/gram	<150 mg/gram

## Data Availability

Any requested data will be made available on request.

## References

[1] Griffith D. E., Aksamit T., Brown-Elliott B. A. (2007). An official ATS/IDSA statement: diagnosis, treatment, and prevention of nontuberculous mycobacterial diseases. *American Journal of Respiratory and Critical Care Medicine*.

[2] Stevens L. A., Padala S., Levey A. S. (2010). Advances in glomerular filtration rate-estimating equations. *Current Opinion in Nephrology and Hypertension*.

[3] Sinniah R. (1983). Occurrence of mesangial IgA and IgM deposits in a control necropsy population. *Journal of Clinical Pathology*.

[4] Manika K., Tasiopoulou K., Vlogiaris L. (2013). Rifampicin-associated acute renal failure and hemolysis: A rather uncommon but severe complication. *Renal Failure*.

[5] De Vriese A. S., Robbrecht D. L., Vanholder R. C., Vogelaers D. P., Lameire N. H. (1998). Rifampicin-associated acute renal failure: Pathophysiologic, immunologic, and clinical features. *American Journal of Kidney Diseases*.

[6] Chen C.-Y., Chen H.-Y., Chou C.-H., Huang C.-T., Lai C.-C., Hsueh P.-R. (2012). Pulmonary infection caused by nontuberculous mycobacteria in a medical center in Taiwan, 2005–2008. *Diagnostic Microbiology And Infectious Disease*.

[7] T Hoen E. F., Hogerzeil H. V., Quick J. D., Sillo H. B. (2014). A quiet revolution in global public health: The world health organization's prequalification of medicines programme. *Journal of Public Health Policy*.

[8] Berthelot P. (1978). Isoniazid-rifampicin: an exemplary hepatotoxicity. *Gastroentérologie Clinique et Biologique*.

[9] Chang C.-H., Chen Y.-F., Wu V.-C. (2014). Acute kidney injury due to anti-tuberculosis drugs: A five-year experience in an aging population. *BMC Infectious Diseases*.

[10] Covic A., Goldsmith D. J. A., Segall L. (1998). Rifampicin-induced acute renal failure: A series of 60 patients. *Nephrology Dialysis Transplantation *.

[11] Muthukumar T., Jayakumar M., Fernando E. M., Muthusethupathi M. A. (2002). Acute renal failure due to rifampicin: A study of 25 patients. *American Journal of Kidney Diseases*.

[12] Poole G., Stradling P., Worlledge S. (1971). Potentially Serious Side Effects of High-Dose Twice-Weekly Rifampicin. *British Medical Journal*.

[13] Park J. T., Lee S., Kim W., Park S. K., Kang K. P. (2017). A Case of Acute Tubulointerstitial Nephritis Associated with Rifampin Therapy Presenting as Fanconi-like Syndrome. *Chonnam Medical Journal*.

[14] Park D. H., Lee S. A., Jeong H. J., Yoo T.-H., Kang S.-W., Oh H. J. (2015). Rifampicin-induced minimal change disease is improved after cessation of rifampicin without steroid therapy. *Yonsei Medical Journal*.

[15] Khalighi M. A., Henriksen K. J., Chang A., Meehan S. M. (2015). Intratubular hemoglobin casts in hemolysis-associated acute kidney injury. *American Journal of Kidney Diseases*.

[16] Kwon S. H., Kim J. H., Yang J. O., Lee E., Hong S. Y. (2004). Ethambutol-induced acute renal failure. *Nephrology Dialysis Transplantation *.

[17] Aouam K., Chaabane A., Loussaïef C., Ben Romdhane F., Boughattas N.-A., Chakroun M. (2007). Adverse effects of antitubercular drugs: epidemiology, mechanisms, and patient management. *Médecine et Maladies Infectieuses*.

